# The role of luck in the success of social media influencers

**DOI:** 10.1007/s41109-023-00573-4

**Published:** 2023-07-25

**Authors:** Stefania Ionescu, Anikó Hannák, Nicolò Pagan

**Affiliations:** Department of Informatics, University of Zurich, Zurich, Switzerland

**Keywords:** Social networks, Recommender systems, Popularity biases, Algorithmic fairness, Agent-based modeling, Markov chains

## Abstract

**Motivation:**

Social media platforms centered around content creators (CCs) faced rapid growth in the past decade. Currently, millions of CCs make livable incomes through platforms such as YouTube, TikTok, and Instagram. As such, similarly to the job market, it is important to ensure the success and income (usually related to the follower counts) of CCs reflect the quality of their work. Since quality cannot be observed directly, two other factors govern the network-formation process: (a) the *visibility* of CCs (resulted from, e.g., recommender systems and moderation processes) and (b) the *decision-making process* of seekers (i.e., of users focused on finding CCs). Prior virtual experiments and empirical work seem contradictory regarding fairness: While the first suggests the expected number of followers of CCs reflects their quality, the second says that quality does not perfectly predict success.

**Results:**

Our paper extends prior models in order to bridge this gap between theoretical and empirical work. We (a) define a parameterized recommendation process which allocates visibility based on popularity biases, (b) define two metrics of individual fairness (ex-ante and ex-post), and (c) define a metric for seeker satisfaction. Through an analytical approach we show our process is an absorbing Markov Chain where exploring only the most popular CCs leads to lower expected times to absorption but higher chances of unfairness for CCs. While increasing the exploration helps, doing so only guarantees fair outcomes for the highest (and lowest) quality CC. Simulations revealed that CCs and seekers prefer different algorithmic designs: CCs generally have higher chances of fairness with anti-popularity biased recommendation processes, while seekers are more satisfied with popularity-biased recommendations. Altogether, our results suggest that while the exploration of low-popularity CCs is needed to improve fairness, platforms might not have the incentive to do so and such interventions do not entirely prevent unfair outcomes.

## Introduction

Over the past few decades, social media platforms have significantly influenced our lives by shaping the information we receive (Bakshy et al. 2012) and the opinions we form Hall et al. (2018). These platforms have shifted from connecting real-life friends to encouraging users to follow strangers based on their content. Today, platforms such as YouTube, Twitter, Instagram, and TikTok are heavily centered around User Generated Content (UGC) and use recommender systems to facilitate the exploration of content.

As a result of this shift, certain users focus on producing content that is semi-professional in nature, which is intended to draw in a growing number of followers and generate income based on their viewership. Given this scenario, it is reasonable to expect that, similarly to the job market (Adams 1963), these social media platforms would ensure fairness for content creators (CCs), where equally qualified individuals are rewarded equally in terms of their visibility, audience, and ultimately their earnings.

To ensure fairness for content creators (CCs) on these online platforms, it is appropriate to ask whether recommender systems produce fair outcomes. In a model proposed by Pagan et al. (2021), each CC has an intrinsic quality. Repeatedly, one CC is recommended to each user; then, users follow the recommended CC when this CC has higher quality than their current followees. This simplified setup thus models one community characterized by a single predominant ordinal attribute which can be perfectly observed by all users (e.g., a community of advanced chess players who want to improve the quality of their game by following the CC most clearly presenting advanced techniques). Simulating this model shows that the expected number of followers for CCs follows a Zipf’s law Zipf (2016), and the expected rankings by quality and followers are the same.

Although the findings of Pagan et al. (2021) imply that UGC-centered platforms are equitable for content creators (CCs), some empirical evidence suggests otherwise. For instance, in cultural markets, predicting success can be challenging (De Vany 2003). According to the experimental study conducted by Salganik et al. (2006), this is partly due to social influence. Specifically, as users receive more information about the prior choices of others, the predictability of an item’s popularity decreases. This lack of predictability can be interpreted as a failure to ensure fairness on certain UGC-centered platforms.

The two lines of work appear thus to be in stark contrast, which raises the question of the underlying cause for the gap in results. While the unidimensionality of the attribute space of CCs and the resulting simplicity of the decision-making process of users could be one cause, we believe that the apparent inconsistency of the previous literature is mostly because of two other limitations in the model used by Pagan et al. (2021). Firstly, the analysis is confined to only two exploratory recommendation processes that use either popularity-based (mimicking the Preferential Attachment mechanism (Barabási and Albert 1999)) or uniformly random (UR) recommendations. However, these simplified recommender systems are limited in their ability to exert social influence and take risks to discover better options, and real-world recommender systems can be different. For instance, recent research has shown the importance of striking the right balance between exploration and exploitation to encourage diversity in recommendation systems (McNee et al. 2006; Kunaver and Požrl 2017; Helberger et al. 2018; Gravino et al. 2019). The second limitation of the model (Pagan et al. 2021) is that they only consider the *expected* number of followers at convergence. However, this ex-ante fairness does not necessarily imply ex-post fairness. Even if content creators receive followers proportional to their quality in expectation, many actual outcomes could still be unfair for at least some unlucky content creators. (Myerson 1981) explains this concept. Additionally, as noted by the authors themselves, there might be long times to convergence, which means that even if a fair outcome is eventually reached, it might not happen within a reasonable time frame.

We address these two challenges by bringing together concepts from network science, recommender systems, and algorithmic fairness. First, we extend the recommendation process beyond UR and the prior implementation of PA by using a parameter $\alpha$ which governs the level of popularity bias in the visibility of CCs. Besides a more granular understanding of various PA-like recommendation processes, this parametrization allows us to also (a) investigate the potential of interventions which increase the visibility of unpopular CCs to improve fairness (i.e., negative values of $\alpha$ which lead to anti-PA recommendation processes (Pollner et al. 2005; Stoikov and Wen 2021)) and (b) investigate extreme versions which recommend only the most (or least) popular CCs (i.e., $\alpha = \pm \infty$, processes which were inspired by the popularity recommender system (Chaney et al. 2018)). Second, we formulate ex-ante and ex-post fairness metrics for CCs, as well as a measure for user satisfaction. Third, we use Markov Chains to theoretically analyze the network formation process under the different recommendation processes and its fairness at convergence. Finally, we use simulations to better investigate how popularity biases and time constraints affect the fairness of CCs and the satisfaction of seekers. Altogether, our work questions whether a unanimous agreement of users on the relative ranking of CCs in terms of desirability is enough to guarantee individual fairness for CC in the final outcome, or if luck still plays an important role.^1^

## Related work

### Network formation

After the seminal work on the random graph model (Erdös and Rényi 1959), the complex networks community began developing straightforward yet insightful mechanisms to explain the emergence of social networks. For instance, the small-world network model (Watts and Strogatz 1998) and the preferential attachment model (PA) (Barabási and Albert 1999) have been used to study the formation of social networks. In the PA model, newborn nodes form connections to existing nodes with a probability proportional to their degree, leading to a rich-get-richer phenomenon where popular nodes become even more popular due to their high visibility. However, the PA model doesn’t emphasize the socio-economic reasons that motivate individuals to form certain connections.

In contrast, some research in sociology (Stochastic Actor Oriented Models (Snijders 1996)) and economics (strategic network formation models (Jackson 2010)) has taken a utilitarian perspective, where agents form connections to maximize some benefit, such as their network centrality. The quality-based model of Pagan et al. (2021) combines both of these approaches by using a user-based ranking system (UR) or PA-based ranking system and a utilitarian decision-making function for users.

To gain a better understanding of how ranking systems and human behavior interact, we introduce a non-exploratory ranking system and study the fairness of the resulting outcomes.

### Fairness

Scholars are not solely concerned with the average performance of processes, but also with their equity in terms of their impact on individuals. As such, much effort has been devoted to developing fairness measures, as well as a methodology for selecting the most appropriate measure depending on the application domain (Verma and Rubin 2018; Garg et al. 2020; Mitchell et al. 2021). Among the various fairness metrics, our focus is on *individual fairness*, which evaluates the extent to which similarly qualified individuals receive similar quality outcomes (see (Binns 2020) for a comprehensive overview of its significance and its apparent incompatibility with other fairness metrics).

A crucial phenomenon in this area is the *timing effect*, which suggests that it is not enough to specify a welfare function; the time at which the function is measured (ex-ante or ex-post) also matters (Myerson 1981). Building on these concepts, we define and examine both ex-ante and ex-post individual fairness for content creators (CCs).

### Recommender systems

In recent years, the recommender system (RS) community has recognized the significance of evaluating RSs beyond accuracy (McNee et al. 2006). Efforts have been made to develop diverse RSs (Kunaver and Požrl 2017; Helberger et al. 2018; Gravino et al. 2019) that ensure any two items can be jointly recommended to users (Guo et al. 2021). This could explain why popularity-based algorithms implemented within the RS community (Chaney et al. 2018; Lucherini et al. 2021) differ from PA (Barabási and Albert 1999; Pagan et al. 2021) by not allowing for a full exploration of recommendations. Our interest in examining extreme PA is motivated by this background, but our primary objective is to understand the network formation process and its fairness. We also differentiate our work from the experimental study of Salganik et al. (2006) as we (a) formally define fairness metrics, (b) use theoretical tools to explain why social influence reduces fairness, and (c) examine network-specific metrics such as the time to convergence.

## Model and metrics

As discussed before, the apparent gap between the conclusions of prior simulation (Pagan et al. 2021) and empirical (Salganik et al. 2006) work regarding the individual fairness of the system could have various roots. On the one hand, it could be caused by the modeling limitations of the recommendation processes. On the other hand, it could be due to how and when fairness is measured. To better understand the degree at which the quality of content creators (CCs) is reflected in their success, we thus need to extend prior models and metrics used to evaluate fairness (Pagan et al. 2021). This section will address each of these extensions in turn To preserve the simplicity of the model, which is critical for its interpretability, we keep most of the simplifying assumptions made in prior work (Pagan et al. 2021; Ionescu et al. 2023).

### Model overview

The platform is composed of two types of users: $n\ge 2$ *content creators* (CCs) who generate content for platforms, and *m* regular users who seek valuable content and will be called *seekers*. Consistent with prior work and observations, we assume there are far more many seekers than CCs. Moreover, we assume CCs are ranked by their quality ($CC_1\succeq CC_2\succeq \dots \succeq CC_n$), and all users prefer higher ranked CCs. In particular, the quality associated with a content creator is a mono-dimensional ordinal attribute^2^ which ultimately induces the same preference ranking of seekers over CCs. Therefore, as done in prior work (Pagan et al. 2021; Ionescu et al. 2023), we model a community of experienced users who all agree on the relative evaluation of CCs, e.g., advanced chess players who want to improve the quality of their game by following the content creator most clearly presenting advanced techniques. This simplifying assumption allows for increased interpretability of the process and its results, as well as for a clear notion of what outcomes are individually fair for CCs. Perhaps most important, it lets us study the effect of the noise within the system (i.e., the luck of CCs) in isolation from other factors (e.g., the size of the target audience).

**Fig. 1:**
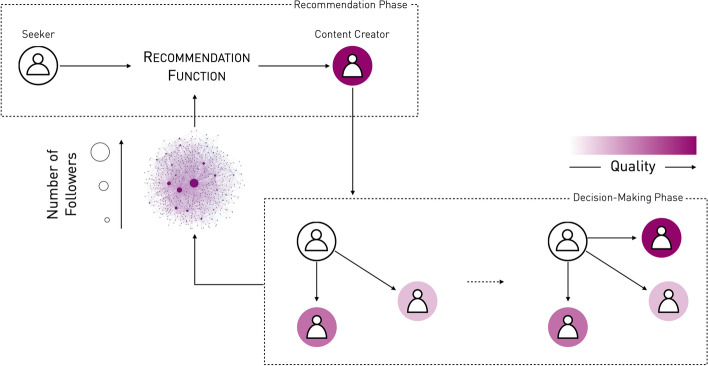
The network formation process is split into two phases: in the first phase, each seeker is recommended a content creator. The probability of recommending a given content creator is based on their current number of followers, as well as on the recommendation process, as shown in 2. In the second phase, the seeker decides whether or not to follow the recommended content creator. This decision process is based on the quality of the recommended content creator and on the quality of the seeker’s current followees: if the quality is greater (or equal) the seeker follows the newly recommended content creator. The two processes are coupled as the decision-making phase changes the status of the network by affecting the number of followers of each content creator. In turn, this changes the probability a given content creator will be recommended

The network formation is a sequential dynamic process where (a) the follower network is initially empty, and (b) at each timestep, each seeker is recommended a CC which they can follow or not. We therefore start with an empty follower network and add edges between seekers and CCs, thus maintaining its bipartite structure 3. As shown in Fig. 1, in each timestep, there is a *recommendation phase* (where each seeker is recommended a CC) and a *decision phase* (where seekers decide whether or not to follow the recommended CC). Seekers follow a recommended CC only if the CC is higher ranked with respect to quality than each of their current followees.

Before explaining the recommendation phase, we introduce some useful notation. Throughout the paper we denote the follower network at time *t* by $A^t\in \{0,1\}^{m\times n}$. As usual, $a^t_{s, c}$ is 1 if seeker $s\in \overline{m}$ follows CC $c\in \overline{n}$ at time *t* and 0 otherwise, where $\overline{k}$ denotes the set of non-zero natural numbers that are at most equal to *k*, i.e. $\overline{k} := \{1, 2, \dots k\}$.

**Fig. 2:**
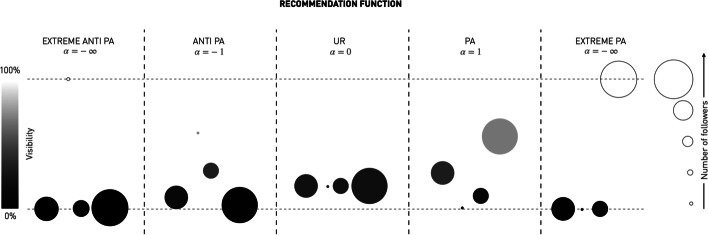
The effect of the different recommendation functions on the visibility of the content creators. Uniform ($\alpha =0)$ provides equal visibility, independently of the number of followers. PA ($\alpha =1$) and extreme PA ($\alpha = \infty$) enhance the visibility of already popular content creators. Vice-versa, antiPA ($\alpha = -1$) and extreme anti PA enhance the visibility of unpopular content creators

*The recommendation phase.* During the recommendation phase, each seeker receives a suggested CC, based on the state of the network. Formally, suggestions are generated depending on the *recommendation process* which maps a follower network $A^t$ to a *recommendation function*, i.e., a function $R^t: \overline{m} \rightarrow \overline{n}$ which maps seekers to CCs 4. The recommendation process we consider here uses the popularity of CCs to distribute their visibility:

$$\begin{aligned} {\mathbb {P}}(R^t_{\alpha } (s) = i) = \frac{(1+a^t_{., i})^{\alpha }}{ \sum _{j\in \overline{n}} (1+a^t_{., j})^{\alpha }}, \end{aligned}$$

where $a_{., i} := \sum _{s\in \overline{m}} a_{s, i}$ is the number of followers of $CC_i$. We introduce this function as a parametrized way of expressing multiple fundamental network formation processes. Positive values of $\alpha$ correspond to preferential attachment (PA), when visibility is proportional to popularity. In particular, when $\alpha =1$ we obtain the same version of PA as in prior work (Pagan et al. 2021; Ionescu et al. 2023). When $\alpha = 0$ we get uniform random (UR) recommendations which distribute visibility equally among CCs. Negative values of $\alpha$ are for anti-preferential attachment (antiPA) (Pollner et al. 2005; Stoikov and Wen 2021) and were not explored in the aforementioned work. Such recommendation processes promote the CCs with fewer (rather than more) followers. Moreover, as we will show later, extreme values of $\alpha$ of plus (minus) infinity 5 correspond to extreme versions of recommendation processes where only the CCs with the most (least) number of followers is recommended. For example, we later show that $\alpha = \infty$ corresponds to $\text {ExtremePA}$ (Ionescu et al. 2023). Figure 2 considers one example of follower counts for CCs and illustrates the recommendation functions resulted from recommendation processes with different values of $\alpha$.

From the perspective of recommender systems (RS), extreme values of $\alpha$ are directly linked to the level of item *availability* (Dean et al. 2020). Similar to prior work, we say content creator $CC_i$ is *reachable* by seeker *s* at time *t* if $CC_i$ can be recommended to *s* at that time step, i.e., if ${\mathbb {P}}(R_\alpha ^t(s) = i) > 0$ 6. Moreover, we say a recommendation process guarantees *complete availability* of CCs to seekers if all content creators are reachable at any timestep. For example, when $\alpha = \pm \infty$, some CCs will eventually become unreachable, meaning those recommendations do not guarantee complete availability. More details will follow in the Theoretical Results Section.

In addition, high values of $\alpha$ correspond to relying more on current CC-popularity resulted from historical interactions, which leads to popularity biases (Bellogín et al. 2017; Abdollahpouri 2019). Reducing the value of $\alpha$ could thus be perceived similarly to increasing the *exploration* of RSs, a change which is thoroughly considered in the RS community (McNee et al. 2006; Kunaver and Požrl 2017; Helberger et al. 2018; Gravino et al. 2019).

We use $(A^t_\alpha )_{t\ge 0}$ to denote our stochastic process. It thus starts from the initial network $A^0$ and sequentially makes recommendations based on the parameter $\alpha$.

### Metrics of interest

As we will see later in the results section, the process described above is a Markov Chain, as the network at the next timestep $A^{t+1}$ only depends on the current network $A^t$. Using this interpretation, we investigate whether the process is an *absorbing* Markov Chain, i.e., if it will eventually reach an *absorbing state* where the network will not change for any possible recommendations. If so, we want to find out the expected number of timesteps until convergence and investigate the fairness in the absorbing states. Doing so allows for comparable results with prior work (Pagan et al. 2021) and a deeper understanding of the causes of the emergent results.

As mentioned in the introduction, we expand our notion of outcome desirability by looking at individual fairness for CCs both in expectation and in the realized outcome. Formally, we define:

- *Ex-post individual fairness for CCs.* We say that a network *A* is *(individually) fair* if the ranking of CCs by quality and popularity are the same; i.e., if $a_{., 1} \ge a_{., 2} \ge \dots \ge a_{., n}$. In particular, we say that an outcome *A* is fair for $CC_i$ if $CC_i$ is one of the top *i* most popular CCs; i.e. if $|\{j: a_{., j} > a_{., i}\}| < i$. We call such outcomes *(individually)* $CC_i$-*fair*. Note that an outcome is fair if it is *CC*-fair for all CCs 7.
- *Ex-ante individual fairness for CCs.* When the resulting process is an absorbing Markov Chain, we can evaluate the fairness of the network formation process by considering the expected number of followers of CCs at convergence. We then say the process is *ex-ante (individually) fair* if the expected number of followers of CCs at absorption is decreasing with respect to their quality index, i.e., if ${\mathbb {E}}[a^{\infty }_{., 1}] \ge {\mathbb {E}}[a^{\infty }_{., 2}] \ge \dots \ge {\mathbb {E}}[a^{\infty }_{., n}]$. Similarly, we say that a process is *ex-ante (individually)* $CC_i$*-fair* if $|\{j: {\mathbb {E}}[a^{\infty }_{., i}]< {\mathbb {E}}[a^{\infty }_{., j}]\}| < i$.

The probability of achieving an ex-post fair outcome for a CC thus corresponds to the chance that their number of followers reflects the quality of their content. As a result, ex-post fairness can be interpreted as an inverse measure of the role of luck, where low values mean that the CC in question needs to be extremely lucky in order to be treated fairly within this system. By defining ex-post fairness for each CC, we can find out which CCs have better chances of reaching fair outcomes at convergence. Moreover, as found previously (Pagan et al. 2021; Ionescu et al. 2023), the time to reach convergence varies a lot depending on the recommendation process. As such, during the simulation results, we also investigate the fairness of the network after a fixed number of timesteps.

As an extension to prior work (Ionescu et al. 2023), we look at how different recommendation processes affect the satisfaction of seekers. Doing so is important in understanding whether changes in the recommendation process could harm the satisfaction of regular users and could thus make that change unappealing to platforms. We measure the dissatisfaction of a seeker based on the quality-wise ranking of the best CC followed by the respective seeker. More precisely, the dissatisfaction of seeker *s* in a network *a* is $\min \{i | a_{s, i} = 1\}$. Therefore, similarly to prior definitions (Jiang et al. 2015; Su 2003), longer search times for the best CC are associated with more dissatisfaction from seekers.

## Theoretical results

Building on prior sections, we use theoretical analysis to better understand the role the recommendation processes play in deciding what types of outcomes we observe. In order, we (i) show our system can be viewed as an absorbing Markov Chain (MC), (ii) investigate the expected time to absorption, and (iii) analyze the fairness of outcomes. To aid understanding, we start each subsection with a paragraph summarizing the results followed by a paragraph which discusses the main take-aways of these results. Afterwards, we proceed to present the theorems and proofs. As a rule, within proofs we aim to only include the details which we believe to be either relevant for understanding the system or more difficult to prove. Details or straightforward steps are thus omitted.

### An absorbing Markov chain

**Fig. 3:**
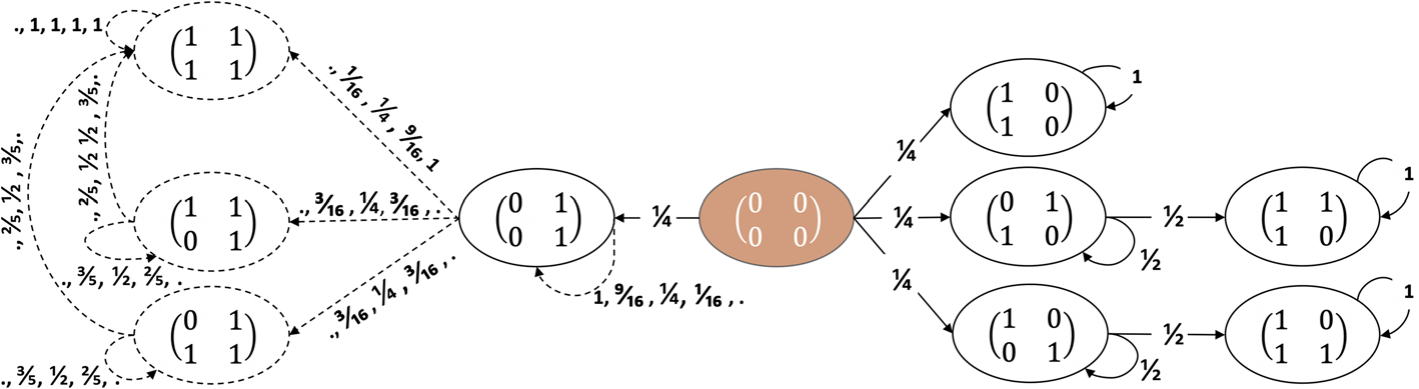
The states and transition probabilities for two users and two CCs. The starting state is the node with the zero matrix (colored in orange). We use full edges when transition probabilities are equal for any value of $\alpha$. When transitions depend on $\alpha$ we use dotted edges. Labels represent the transition probabilities for (1) ExtremePA ($\alpha = \infty$), (2) PA with $\alpha = 1$, (3) UR ($\alpha = 0$), (4) AntiPA with $\alpha = -1$, and (5) ExtremeAntiPA ($\alpha = - \infty$) respectively. Dots replace zeros or probabilities when the starting state is not reachable from **0** under the respective recommendation process

*Summary.* Cornerstone to our paper is the view of the process as an absorbing Markov Chain (MC). We thus start with Theorem 1 which proves such a view is correct. To better understand the process, we depict in Fig. 3 the states and transitions for the small case of two seekers and two content creators (CCs) under different parameters for the recommendation process. We use the rest of the subsection to characterize the transitions and the absorbing states. Lemma 1 proves that for finite values of $\alpha$ availability is guaranteed, while for plus (minus) infinity, only CCs with the highest (lowest) number of followers can be recommended. This means that the absorbing states are very different depending on the recommendation process. Theorem 2 shows that when availability is guaranteed (i.e., when $\alpha \ne \pm \infty$) the absorbing states are the ones where each user follows the best CC. For the remaining recommendation processes (i.e., the extreme versions when $\alpha = \pm \infty$), a state is absorbing if and only if no additional seeker would follow any of the CCs with the *highest* (when $\alpha = \infty$ or *least* when $\alpha = - \infty$) number of followers (see Theorem 3). Moreover, using Lemma 2, we prove that for ExtremePA (i.e., $\alpha = \infty$) all absorbing states reachable form ${\textbf {0}}$ have in addition a unique most followed CC.

*Take-away.* The results of this subsection build a representation of the process, which facilitates its understanding. Specifying the transition matrix allows us to see the impact of popularity biases within recommendation processes. When availability is not guaranteed, we have more absorbing states. Among these, only the states where all seekers found the best CC (i.e., $CC_1$-fair ones) are also absorbing under availability. Moreover, PA lies between UR and Extreme PA in terms of exploration: As we rely increasingly more on popularity (i.e., increasing the value of $\alpha$) the chance of remaining in transient states (which are fairness-wise similar to the states which are absorbing only under ExtremePA) grows. Finally, all these results are key in understanding the time to convergence and fairness which we investigate in the upcoming subsections.

#### Theorem 1

$(A^t_\alpha )_{t\ge 0}$ is an absorbing MC (for any value of $\alpha$).

#### Proof

By our modeling of the network formation process, the configuration of the network at the next timestep depends only on the current state of the network and not on the history. Formally, $(A^t_\alpha )_{t\ge 0}$ is a MC with: (a) $\{0, 1\}^{m\times n}$ as the state space, (b) $\lambda$ (where $\lambda _{A} = 1$ iff $A = {\textbf {0}}$) as the initial distribution, and (c) $p_{B, C} := {\mathbb {P}}(A^{t+1} = C|A^t = B)$ as the transition matrix. For the latter, we transit only to states where each seeker either (1) follows the same CCs as before, or (2) follows exactly one more CC which is better than the best CC they followed so far (i.e., for all seekers *s* there exists at most one $CC_i$ such that $b_{s, j} = 0\ \forall \ j\le i$ and $c_{s, i} = 1$). The exact probabilities of such transitions generally depend on the value of $\alpha$. The only exception is when all CCs have the same number of followers. In particular, from the initial state $A^0 = {\textbf {0}}$ we can only transit to a network where each seeker follows precisely one CC:

$$\begin{aligned} p_{{\textbf {0}}, C} = \left\{ \begin{array}{ll} 1/{n^m}, &{} \text {if } c_{s, .} = 1 \ \forall \ s \in \overline{m} \\ 0, &{} \text {otherwise}\\ \end{array} \right. \end{aligned}$$

Finally, based on the monotonous properties of the transition matrix we can show that $(A^t_\alpha )_{t\ge 0}$ is absorbing: Since $p_{B, C}$ is non-zero iff $C=B$ or there is some user *u* who follows one more CC, any state *B* is either absorbing or can transit to a state of a strictly higher sum of elements. However, the sum of elements of any state is bounded above by the number of entries (i.e., $m \cdot n$). Hence, such a sequence of transitions must be finite and it eventually reaches an absorbing state. $\square$

#### Lemma 1

When $\alpha$ is finite all recommendation process guarantee complete availability for all seekers. Differently, when $\alpha = \pm \infty$, only CCs with the highest (lowest) number of followers have a nonzero probability to be recommended. Thus availability is no longer guaranteed for $\alpha = \pm \infty$.

#### Proof

The first statement follows directly from the definition of the recommendation process: when $\alpha \in {\mathbb {R}}$, ${\mathbb {P}} (R_\alpha ^t(s) = i) > 0$ for any follower matrix $A^t$. Therefore, each $CC_i$ is reachable for all seekers *s*. By definition, availability is thus guaranteed.

When $\alpha = \infty$, however, CCs that do not have the maximum number of followers are inaccessible to seekers:

$$\begin{aligned} {\mathbb {P}}(R^t_{\infty } (s) = i)&= \lim _{\alpha \rightarrow \infty } \frac{(1+a^t_{., i})^{\alpha }}{ \sum _{j\in \overline{n}} (1+a^t_{., j})^{\alpha }} \\&= \lim _{\alpha \rightarrow \infty } \frac{1}{ \sum _{j\in \overline{n}} \left( \frac{1+a^t_{., j}}{1+a^t_{., i}}\right) ^{\alpha }} \\&= \left\{ \begin{array}{ll} 1/{|\arg \max _j a^t_{., j}|}, &{} \text {if } i\in \arg \max _j a^t_{., j} \ \forall \ s \in \overline{m} \\ 0, &{} \text {otherwise}\\ \end{array} \right. \end{aligned}$$

where $\arg \max _j a_{., j}$ is the set of CCs with the maximum number of followers in *A*. Analogous results hold when $\alpha = -\infty$. So, availability is not guaranteed for extreme recommendation processes. $\square$

#### Theorem 2

When $\alpha$ is finite, a state *B* is absorbing iff all users follow the best CC, i.e. iff $b_{s, 1} = 1$ for all $s\in \overline{m}$.

#### Proof

($\Rightarrow$) We prove the direct implication by contradiction. Let *B* be a state with $b_{s, 1} = 0$ for some seeker *s*. By Lemma 1, $CC_1$ is available to *s*. So, *s* can first receive $CC_1$ as a recommendation and thus follow them. Hence, we have a non-zero probability of transitioning from *B* to a state $C\ne B$ where $c_{s, 1} = 1$. In particular, it implies *B* is not absorbing.

($\Leftarrow$) For the converse, assume $b_{s, 1} = 1$ for all seekers *s*. Since everybody already found the best CC, no seeker will follow somebody new. This means that no recommendations will change the follower network, thus making *B* absorbing. $\square$

#### Theorem 3

When $\alpha = \infty$ (or $\alpha = -\infty$), a state is absorbing iff every seeker follows a CC at least as good as the highest-quality CC with the maximum (minimum) number of followers. Formally, when $\alpha = \pm \infty$, *B* is absorbing iff for all seekers *s* there exists some $j\le e_\alpha (B)$ s.t. $b_{s, j} = 1$, where $e_\infty (B) = \min \arg \max _i b_{., i}$ and $e_{-\infty }(B) = \min \arg \min _i b_{., i}$

#### Proof

($\Rightarrow$) Assume *B* is absorbing, but there exists some seeker *s* who does not follow anybody at least as good as $e_\alpha (B)$; i.e., $b_{s, i} = 0$ for all $i \le e_\alpha (B)$. By Lemma 1, $e_\alpha (B)$ has a nonzero probability of being recommended to *s*. Therefore, from *B* we can transit to a new network *C* where $c_{s, e_\alpha (B)} = 1$. Thus, we reach a contraction as *B* is not absorbing.

($\Leftarrow$) If all seekers follow somebody at least as good as the highest-quality *CC* with the maximum (minimum) number of followers, then no seeker will follow a CC with the maximum (minimum) number of followers if recommended. However, by Lemma 1, these CCs are the only ones who have a chance of being recommended when $\alpha = \pm \infty$. Thus, *B* is absorbing. $\square$

#### Lemma 2

Any state reachable from **0** has, for each $CC_i$ that has the maximum number of followers, a seeker *s* who does not follow any CC better than $CC_i$. I.e., if $A^0 = {\textbf {0}}$ then for any $t\in {\mathbb {N}}$ and $i\in \overline{n}$ s.t. $a^t_{., i} = \max _j a^t_{., j}$ there exits some seeker $s \in \overline{m}$ s.t. $a^t_{s, j} = 0$ for all $j<i$.

#### Proof

We prove this by induction on *t*. After the first timestep (i.e., when $t=1$) each seeker follows precisely one CC, so the claim holds.

Assume the claim is true at time *t* and let $M^t = \arg \max _j a^t_{., j}$ be the set of the CCs with the maximum number of followers at time *t*. From $A^t$, we can either return to the same state (i.e., $A^{t+1} = A^t$) or we transit to a different network where a subset of the CCs who originally had the maximum number of followers still do (i.e., $M^{t+1} \subset M^t$). In the first case, the claim trivially continues to hold. In the latter case, each $CC_i$ with $i\in M^{t+1}$ increased their number of followers between the two timesteps. Therefore, each such $CC_i$ was recommended to at least one seeker *s* who decided to follow them. This makes $CC_i$ the best CC *s* follows in $A^{t+1}$, i.e., $i = \min \{j: a^{t+1}_{s, j} = 1\}$. Hence, the claim. $\square$

#### Corollary 1

When $\alpha = \infty$, a state reachable from **0** is absorbing iff it has (a) a unique most followed CC, and (b) all users follow either this most followed CC or a better one. I.e., if $A^0 = {\textbf {0}}$ then $A^t$ is absorbing iff (a) there exist some $i \in \overline{n}$ s.t. $a^t_{., i}> b_{., j}$ for all $j \in \overline{n}-\{i\}$, and (b) for all $s\in \overline{m}$ there exist some $j\le i$ s.t. $a^t_{s, j} = 1$.

#### Proof

($\Rightarrow$) Assume $A^t$ is absorbing, but two CCs have the highest number of followers, say $CC_i$ and $CC_j$ with $i<j$. By Lemma 2, some seeker *s* does not follow $CC_i$. By Lemma 1, there is a nonzero probability to recommend $CC_i$ to *s* and thus transit to a new state. Thus, $A^t$ is not absorbing (contradiction). So, if $A^t$ is absorbing then (a) must hold. Moreover, (b) must also hold by Theorem 3.

($\Leftarrow$) The converse is an immediate consequence of Theorem 3. $\square$

### Expected time to absorption

**Fig. 4:**
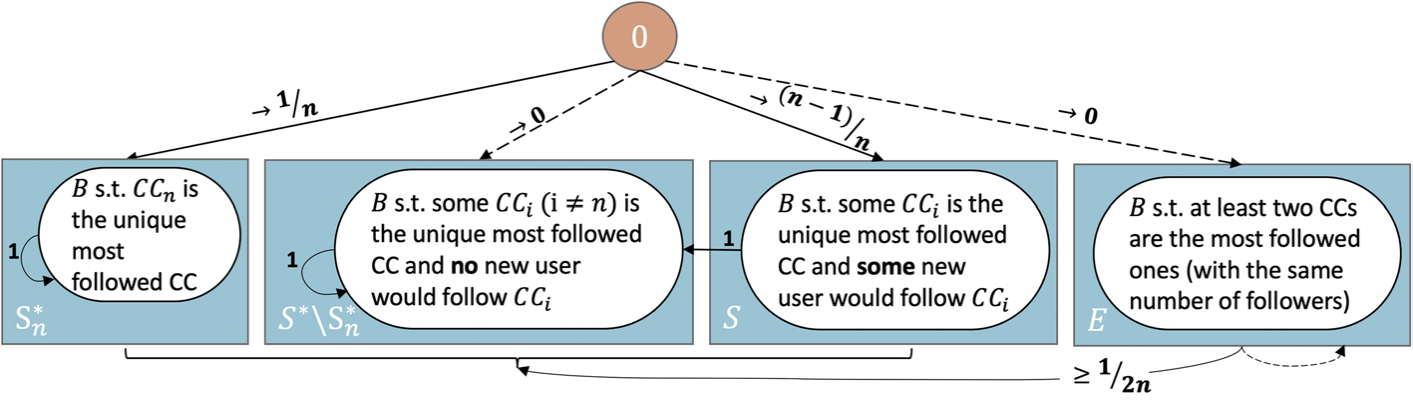
The diagram shows the main states and transitions when $\alpha = \infty$ and $m\rightarrow \infty$. We use rectangles for sets of states and circles for the general form of the states in each set. The annotations for the transition probabilities are in the limit. When these probabilities go to 0 as $m\rightarrow \infty$ we use dotted edges

**Fig. 5:**
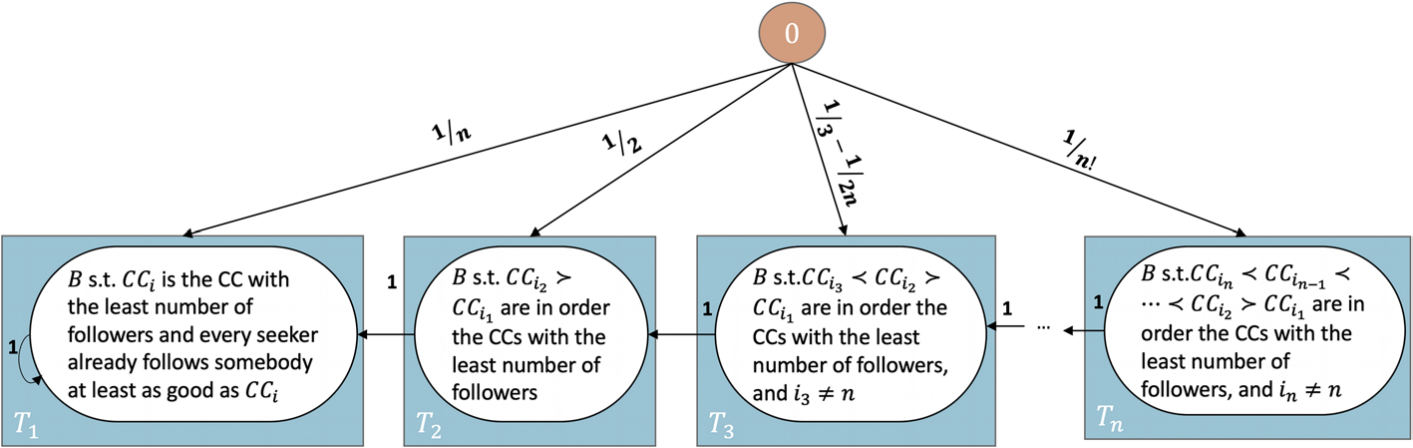
The diagram shows the main states and transitions when $\alpha = - \infty$, given that no two CCs have an equal number of followers after the first timestep. We use rectangles for sets of states and circles for the general form of the states in each set

*Summary.* To investigate the time to absorption under extreme recommendation processes, we first prove some preliminary results. First and most important, the chance of having two content creators (CCs) with an equal number of followers after the first round of recommendations (Lemma 3) goes to zero as the number of seekers grows to infinity. Second, the probability there is no seeker which would follow the most popular $CC_i$ if recommended also goes to 0 as the number of seekers goes to infinity (for $i\ne n$, see Lemma 4)^8^. Third, Lemma 5 and Theorem 5 formalize the following intuition: If, after the first timestep, no two CCs have the same number of followers, then extreme anti-PA will recommend in turn the CCs with the least number of followers until it either recommends $CC_n$ or a CC of a lesser quality than in the previous round. Based on these observations, we obtain most of the annotations in Figs. 4 and 5, figures which summarize the process and provide the intuition for proving the two main results. Theorem 4 shows that when $\alpha = \infty$ the process is expected to converge in $2-1/n$ timesteps. Theorem 6 proves that when $\alpha = -\infty$ the process is almost always expected to converge in about $e\cdot (n-1)/n$ many timesteps.

*Take-away.* This puts extreme recommendation processes in sharp contrast with those guaranteeing availability. While our results show that extreme recommendations generally lead to fast convergence, prior work reveals that Uniform Random (UR) and Preferential Attachment (PA) (with $\alpha = 1$) convergence times increase logarithmically in the number of CCs and linearly (or sub-linearly) in the number of users (see Figs. 2 and 7b of Pagan et al. (2021)). Consequently, while for extreme recommendations it could be sufficient to consider the fairness at convergence, this might not be the case for general values of $\alpha$. Longer convergence times indicate that fairness in transient states has a high relevance. This is particularly true for PA recommendation processes which, as observed in the prior subsection, remain for longer in unfair states. To expand on this, the upcoming simulations will look at fairness at intermediate timesteps.

#### Lemma 3

As the number of seekers grows to infinity, the probability two CCs will have the same number of followers after the first timestep goes to zero. That is, for any $i\ne j \in \overline{n}$, ${\mathbb {P}}(a^1_{., i} = a^1_{., j}) \rightarrow 0$ as $m\rightarrow \infty$.

#### Proof

For each seeker *s*, we define a random variable $Y_s$ based on the recommendations in the first round:

$$\begin{aligned} Y_s = \left\{ \begin{array}{ll} 0, &{} \text {if seeker } s \text { is recommended some } CC_k \text { with } k \ne i, j; \\ 1, &{} \text {if seeker } s \text { is recommended } CC_i;\\ -1, &{} \text {if seeker } s \text { is recommended } CC_j. \end{array} \right. \end{aligned}$$

As, in the first timestep, the recommended CC is chosen uniform randomly, $(Y_s)_{s\in \overline{m}}$ are i.i.d. (with ${\mathbb {P}}(Y_s = 0) = (n-2)/n$ and ${\mathbb {P}}(Y_s = c) = 1/n$ when $c = \pm 1$). Hence, ${\mathbb {E}}[Y_s] = 0$ and $\text {Var}(Y_s) = {\mathbb {E}}[Y_s^2] = 2/n$. By the central limit theorem it follows that,

$$\begin{aligned} \frac{\sum _{s\in \overline{m}} Y_s - m\cdot 0}{2/n \cdot \sqrt{m}} \rightarrow ^{\mathcal {D}} \mathcal {N}(0, 1). \end{aligned}$$

Equivalently, for any constants $c_1 \le c_2$ we have $\lim _{m\rightarrow \infty } {\mathbb {P}}\left( c_1 \le \frac{a^1_{., i} - a^1_{., j}}{2/n \cdot \sqrt{m}}\le c_2 \right) = \int _{c_1}^{c_2} \frac{1}{\sqrt{2\pi }} e^{-\frac{1}{2}x^2} dx$. For $c_1 = c_2 = 0$, this implies that $\lim _{m\rightarrow \infty } {\mathbb {P}}(a^1_{., i} = a^1_{., j}) = 0$. $\square$

#### Lemma 4

The probability a non bottom-quality $CC_i$ is the most (least) followed CC and no seeker would follow $CC_i$, if recommended in the first round, goes to zero as the number of seekers goes to infinity. Formally, for any $i \in \overline{n-1}$, ${\mathbb {P}}( (a^1_{., i} > a^1_{.,j}\ \forall j\in \overline{n}) \wedge (\forall s \in \overline{m},\ \exists j \in \overline{i} \text{ s.t. }\ a^1_{s, j} = 1)) \rightarrow 0$ as $m\rightarrow \infty$. Same for the probability when the first condition in the conjunction is $a^1_{., i} < a^1_{.,j}\ \forall j\in \overline{n}$.

#### Proof

To show this, note that the second condition in the conjunction is equivalent to the case when all seekers were recommended in the first round either $CC_i$ or a better quality CC. The chance that all seekers were recommended one of the top-quality *i* CCs in the first round is $\left( \frac{i}{n} \right) ^m$ and thus goes to zero as the number of seekers go to infinity. So, ${\mathbb {P}}( (a^1_{., i} > a^1_{.,j}\ \forall j\in \overline{n}) \wedge (\forall u \in \overline{m},\ \exists j \in \overline{i}\ a^1_{u, j} = 1)) \le {\mathbb {P}}(\forall u \in \overline{m},\ \exists j \in \overline{i}\ a^1_{u, j} = 1)= \left( \frac{i}{n} \right) ^m \rightarrow 0$ as $m\rightarrow \infty$. The same is true first condition is $a^1_{., i} < a^1_{.,j}\ \forall j\in \overline{n}$. $\square$

#### Theorem 4

When $\alpha = \infty$, the expected time to absorption goes to $2-1/n$ as *m* goes to infinity.

#### Proof

Based on Theorems 1 and Corollary 1, we can group states in subsets, as shown in Fig. 4. Let $\mu _B$ be the expected time from the state *B* to absorption. Then $\mu _{B: B\in S^*} = 0$ (as all states in $S^*$ are absorbing) and $\mu _{B: B\in S} = 1$ (as all states in *S* lead in one timestep to a state in $S^*$). Therefore,

$$\begin{aligned} \mu _{{\textbf {0}}} = 1\cdot p_{{\textbf {0}}, S_n^*} + 1\cdot p_{{\textbf {0}}, S-S_n^*} + 2\cdot p_{{\textbf {0}}, S} + \sum _{B\in E} (1 + \mu _B) \cdot p_{{\textbf {0}}, B} \end{aligned}$$

We can use Lemmas 3 and 4 to find the transition probabilities from **0** to sets of states as $m\rightarrow \infty$: (a) $p_{{\textbf {0}}, S^*_n}\rightarrow \frac{1}{n}$, (b) $p_{{\textbf {0}}, S^*-S^*_n}\rightarrow 0$, (c) $p_{{\textbf {0}}, S}\rightarrow \frac{n-1}{n}$, and (d) $p_{{\textbf {0}}, E} \rightarrow 0$. Moreover, since *E* has finitely many states, one of them has the maximum probability of being realized from ${\textbf {0}}$ (i.e., $\mu _{B^*} = \max _{B\in E} \mu _B$). Then, $\sum _{B\in E} p_{{\textbf {0}}, B} \cdot (1 + \mu _B) \le p_{{\textbf {0}}, E}\cdot (1+ \mu _{B^*})$ and $\mu _{B^*}\le c$ (*c* constant)^9^. Consequently, $\lim _{m\rightarrow \infty } \mu _0 = 1\cdot \frac{1}{n} + 2\cdot \frac{n-1}{n} = 2-\frac{1}{n}$. $\square$

#### Lemma 5

Assume $\alpha = -\infty$ and *B* is a state where (a) $CC_{i_k}, \dots , CC_{i_1}, CC_{i_0}$ are the CCs with the least number of followers (with $b_{., i_k}< \dots< b_{., i_i} < b_{., i_0}$), (b) the number of followers of all $CC_{i_j}$ is pairwise distinct, (c) there are at least $b_{., i_0}$ many seekers who would follow any of $CC_{i_j}$ with $j\ne 0$ and there is no seeker who would follow any CC with an equal number of followers to $CC_{i_0}$ (if any such CC), and (d) $i_k> \dots > i_1$ and $i_1 <i_0$. Then from *B* we transit with probability 1 to a state *C* where the only difference is that $CC_{i_k}$ has at least as many followers as $CC_{i_0}$ and no seeker would follow $CC_{i_k}$. Moreover, if $k=1$ then *C* is an absorbing state.

#### Proof

By assumption (c) there is a set *X* of at least $b_{., i_0}$ seekers who would follow $CC_{i_k}$ if recommended. Since $CC_{i_k}$ is the unique least followed CC, it will be recommended to all seekers and all seekers in *X* will thus follow $CC_{i_k}$. This makes $CC_{i_k}$ have at least as many followers as $CC_{i_0}$. Moreover, since $CC_{i_j}$ has higher quality than $CC_{i_k}$ for all $0<j<k$ (by assumption c) , all seekers in *X* would follow such a $CC_{i_j}$ if recommended. Therefore, from *B* we transit in one timestep to a state where (a) $CC_{i_{k-1}}, \dots , CC_{i_1}, CC_{i_0}$ are the CCs with the least number of followers (with the same relative ordering as none were recommended this turn), (b) the number of followers of all $CC_{i_j}$, $j<k$, is pairwise distinct, (c) there are at least $b_{., i_0}$ many seekers who would follow any of $CC_{i_j}$ with $j\ne 0$ and no one would follow any CC with the same number of followers as $CC_{i_0}$, and (d) $i_{k-1}> \dots > i_1$ and $i_1 <i_0$.

In addition, $CC_{i_1}$ would be recommended before $CC_{i_0}$ and $CC_{i_1}$ has a higher quality than $CC_{i_0}$. Thus, when $CC_{i_0}$ is recommended no new seeker would follow them. Nor would any seeker follow a CC with an equal number of followers with $CC_{i_0}$ (by assumption c and since $CC_{i_1}$ was just recommended). Thus we reached an absorbing state.

$$\square$$

#### Theorem 5

Assume $\alpha = -\infty$ and after one timestep all *CC*s have distinct numbers of followers (say $CC_{i_1}, \dots , CC_{i_n}$ in increasing order of their number of followers). Let *k* be either 1 if $CC_n$ is the least followed CC, or, when this is not the case, the lowest $j>1$ such that $CC_{i_j}$ is of a lesser quality than $CC_{i_{j-1}}$. Then, $(A^t_{-\infty })_{t\ge 0}$ converges in *k* timesteps.

#### Proof

First we note that *k* is well defined as there is always such a lowest *j*. If $k=1$ and $CC_n$ is the unique least followed *CC*, then only $CC_n$ will be recommended but no one new will follow them. So, $(A_t^\infty )_{t\ge 0}$ is absorbing in one timestep.

If $k>1$, $CC_{i_0}$ is the least followed *CC* and $i_j < i_0$ for all $j<k$. Since $i_0 < n$, this means that $CC_n$ is not among the $(k-1)$ least followed CCs. Thus, $CC_n$ has at least as many followers as followers as $CC_{i_{k-1}}$. All the followers of $CC_n$ would follow any of the $CC_{i_j}$ with $j<k$ as every $CC_{i_j}$ has a higher quality than $CC_n$ (thus meeting condition (c) in Lemma 5). The other three conditions in Lemma 5 are trivially true. By the aforementioned lemma, we iteratively transit to new states where $CC_{i_j}$ is the unique least followed CC, where *j* takes in order values from 1 to *k*. When $j = k$, by the same lemma, we reached an absorbing state. Thus $(A^t_{-\infty })_{t\ge 0}$ converged in *k* steps. $\square$

#### Theorem 6

When $\alpha = -\infty$, the expected time to absorption given that no two *CCs* have the same number of followers after the first round is $\frac{n-1}{n}\sum _{k=0}^{n-2} \frac{1}{k!} - \frac{1}{n}\sum _{k=0}^{n-2} \frac{k}{k!} + \frac{1}{n}\sum _{k=0}^{n-1} \frac{1}{k!}$. This is asymptotically equivalent to $\frac{n-1}{n}\cdot e$.^10^

#### Proof

Assume no two CCs have the same number of followers after the first timestep. Let $T_k$ be the set of states where *k* is either 1 if $CC_n$ is the least followed CC, or, when this is not the case, the lowest $j>1$ such that $CC_{i_j}$ is of a lesser quality than $CC_{i_{j-1}}$ (i.e., as in Theorem 5 and Fig. 5).

Now, we find the probabilities of transitioning from ${\textbf {0}}$ to each of these sets, given that no two CCs will have the same number of followers. For $T_1$, $p_{{\textbf {0}}, T_1} = \frac{1}{n}$ since there is a unique least followed CC, and, by symmetry, all *n* CCs have the same chance of being on that position. For $T_k$ with $k>1$:

$$\begin{aligned} p_{{\textbf {0}}, T_k} = \frac{{n-1 \atopwithdelims ()k}\cdot (k-1) + {n-1 \atopwithdelims ()k-1}}{{n \atopwithdelims ()k} \cdot k!} = \frac{(n-k) \cdot (k-1)}{n\cdot k!} + \frac{1}{n\cdot (k-1)!}. \end{aligned}$$

Note that the formula above does, in fact, also hold for $k=1$ (as it gives $p_{{\textbf {0}}, T_1} = 1/n$). Moreover, by Theorem 5, the process converges exactly in *k* steps when it passes through state $T_k$ in the first timestep. As such, the expected time to convergence from ${\textbf {0}}$ given that there is no equality after the first timestep is:

$$\begin{aligned} \mu _{{\textbf {0}}}&= \sum _{k=1}^n k \cdot p_{{\textbf {0}}, T_k}\\&= \sum _{k= 2}^{\infty } \frac{(n-k) \cdot (k-1)\cdot k}{n\cdot k!} + \sum _{k= 1}^{\infty } \frac{k}{n\cdot (k-1)!}\\&= \frac{n-1}{n}\sum _{k=0}^{n-2} \frac{1}{k!} - \frac{1}{n}\sum _{k=0}^{n-2} \frac{k}{k!} + \frac{1}{n}\sum _{k=0}^{n-1} \frac{1}{k!}. \end{aligned}$$

This is asymptotically equivalent to $\frac{n-1}{n}\cdot e$. $\square$

### Fairness for content creators

*Summary.* Based on the results of the previous subsections, Corollary 2 proves that non-extreme recommendation processes are both ex-ante and ex-post fair for the best quality content creator (i.e., $CC_1$-fair). Extreme recommendation processes, on the other hand, are rarely leading to a $CC_1$-fair absorbing state (see Corollaries 3 and  4). However, Theorem 7 shows these processes continue to be ex-ante $CC_1$-fair.

*Take-away.* Together, these results show that availability plays a vital role in the impact of luck on observing a fair outcome for the best content creator (CC), i.e. in the likelihood of observing ex-post $CC_1$-fair outcomes. While recommendation processes that do not guarantee complete availability are ex-ante fair, they are rarely ex-post fair, even for the highest quality CC. This result aligns with the prior experimental work^11^, thus confirming that it is crucial to understand and analyze extreme recommendation processes as they share important similarities with real-life ones.

#### Corollary 2

When $\alpha$ is finite, $(A_\alpha ^t)_t$ is both ex-post and ex-ante $CC_1$-fair.

#### Proof

This is an immediate consequence of Theorem 2. Since in all absorbing states for non-extreme recommendation processes $CC_1$ is followed by all seekers, $CC_1$ always has *m* followers and is, thus, (at least weakly) the most followed CC. So, all absorbing states are $CC_1$-fair (i.e., ex-post fairness) and ${\mathbb {E}}[b_{., 1}] = m \ge {\mathbb {E}}[b_{., i} ]$ for all $i\in \overline{n}$ (i.e., ex-ante $CC_1$-fairness). $\square$

#### Corollary 3

When $\alpha = \infty$, the probability the outcome is ex-post $CC_1$-fair goes to 1/*n* as $m \rightarrow \infty$. More generally, as $m \rightarrow \infty$, the probability of $CC_k$-fairness goes to *k*/*n*, while the probability of ex-post fair outcomes for all CCs goes to 1/*n*!.

#### Proof

For the first part, note that the set of $CC_1$ fair states (a) contains the set of states where $CC_1$ is the unique most followed CC after the first round, and (b) is contained in the set of states where $CC_1$ is one of the CCs with a maximum number of followers after the first round. If, as in Fig. 4, *E* is the set of states where at least two CCs are the most followed and $S_1\cup S_1^*$ is the set of states where $CC_1$ is the unique most followed CC, then $p_{{\textbf {0}}, S_1\cup S_1^*} \le {\mathbb {P}}(CC_1-\text {fair}) \le p_{{\textbf {0}}, S_1\cup S_1^*} + p_{{\textbf {0}}, E}.$ Since, as shown before, $p_{{\textbf {0}}, S_1\cup S_1^*}\rightarrow 1/n$ and $p_{{\textbf {0}}, E}\rightarrow 0$, we conclude that ${\mathbb {P}}(CC_1-\text {fair}) \rightarrow 1/n$ as $m\rightarrow \infty$.

More generally, the set of $CC_k$-fair states (a) contains the set of states where no two CCs have an equal number of followers and $CC_k$ is in the top *k* CCs (according to the number of followers) after the initial round of recommendations, and (b) is contained in the set of states where $CC_k$ is one of the top *k* CCs after the first round of recommendation. Analogous to above, and since, by symmetry, all choices of the top *k* CCs has the same probability of occurring, ${\mathbb {P}}(CC_1-\text {fair}) \rightarrow \frac{{{n-1}\atopwithdelims (){k-1}}}{{{n}\atopwithdelims (){k}}} = \frac{k}{n}$.

Finally, similarly as before, the chance of ties in the number of followers after the first round of recommendations goes to zero as *m* goes to infinity. If no two CCs have the same number of followers, then we achieve ex-post fairness iff, after the first timestep, $a^1_{., 1}> a^1_{., 2}> \dots > a^1_{., n}$. But all *n*! strict orderings of the follower counts after the first round occur with an equal probability (by symmetry). Thus, ${\mathbb {P}}(\text {ex-post fairness}) \rightarrow 1/n!$ as $m\rightarrow \infty$. $\square$

#### Corollary 4

When $\alpha = -\infty$, the probability the outcome is ex-post $CC_1$-fair as $m \rightarrow \infty$ is at most $\frac{1}{n} + \frac{1}{n}\cdot \sum _{k=1}^{n-1} \frac{1}{(k-1)!} - \frac{1}{n(n-1)}\cdot \sum _{k=1}^{n-1} \frac{k-1}{(k-1)!}$. This is asymptotically equivalent to $(e+1)/n$.

#### Proof

Let (dif) be the condition that all CCs have a distinct number of followers. The key observation is that, if after the first timestep:

- (dif) holds and $CC_1$ is the least followed *CC* then $(A^t_{-\infty })_{t\ge 0}$ always converges in the next timestep to a $CC_1$-fair state (since $CC_1$ will be recommended and thus followed by all seekers). We call the set of such states $T_1$;
- (dif) holds and $CC_1$ is the *k*-th most followed *CC* (but not the least or the most followed one) then $CC_1$ will eventually be the most followed *CC* (i.e., we have ex-post $CC_1$-fairness) iff (a) the least most followed *CC*s until $CC_1$ are ordered in increasing order of their quality and (b) $CC_n$ is not the least followed one (by Lemma 5). We call the set of such states $T_k$. Moreover, if the least followed $k-1$ *CC*s are not the bottom or top quality ones and are ordered in increasing order of their quality we say the ordering has property (right);
- (dif) does not hold or (dif) holds and $CC_1$ is the most followed *CC* then $(A^t_{-\infty })_{t\ge 0}$ might be ex-post $CC_1$ fair at convergence. We call *F* the set of states where (dif) does not hold, and $T_n$ the set of states where (dif) holds and $CC_1$ is the most followed *CC*.

Then, $\sum _{k=1}^{n-1} p_{{\textbf {0}}, T_k} \le {\mathbb {P}}(CC_1\text {-fairness}) \le p_{{\textbf {0}}, F} + \sum _{k=1}^{n} p_{{\textbf {0}}, T_k}$. But, if $k<n$

$$\begin{aligned} p_{{\textbf {0}}, T_k}&= (1-p_{{\textbf {0}}, F})\cdot {\mathbb {P}}(CC_1 \text { is the { k}-th least followed}|\text {(diff)})\\&\ \ \ \cdot {\mathbb {P}}( \text {the least followed (k-1) CCs are (right)}|\text {(diff)})\\&= (1-p_{{\textbf {0}}, F}) \cdot \frac{1}{n} \cdot \frac{{n-2\atopwithdelims ()k-1}}{{n-1\atopwithdelims ()k-1} \cdot (k-1)!}\\&= (1-p_{{\textbf {0}}, F}) \cdot \frac{1}{n} \cdot \left( \frac{1}{(k-1)!} - \frac{1}{n-1}\cdot \frac{k-1}{(k-1)!}\right) \end{aligned}$$

Since, by Lemma 3, $\lim _{m\rightarrow \infty } p_{{\textbf {0}}, F} = 0$ and $p_{{\textbf {0}}, T_n}\le (1-p_{{\textbf {0}}, F})\cdot \frac{1}{n}$ it follows that $\lim _{m\rightarrow \infty } {\mathbb {P}}(CC_1\text {-fairness}) \le \frac{1}{n} + \frac{1}{n}\cdot \sum _{k=1}^{n-1} \frac{1}{(k-1)!} - \frac{1}{n(n-1)}\cdot \sum _{k=1}^{n-1} \frac{k-1}{(k-1)!}$. This is asymptotically equivalent to $\frac{e+1}{n}$. $\square$

#### Theorem 7

When $\alpha = \pm \infty$, $(A_\alpha ^t)_{t\ge 0}$ is ex-ante fair.

#### Proof

The proof is based on the following observation: If $X_i$ is the number of seekers who would follow $CC_i$ if recommended after the first round, then $X_1 \supseteq \dots \supseteq X_n$.

For extreme PA, this means that when $CC_i$ becomes the most followed *CC*, they will be eventually followed by all CCs who did not follow a better-quality CC before. For example, if $CC_2$ is the most followed after the first round, all users (except those who were recommended $CC_1$) will follow $CC_2$ after round 2. Differently, if $CC_1$ was the most followed, then everybody will end up following $CC_1$ next, while if $CC_3$ was the most followed, then everybody except those who originally followed $CC_1$ or $CC_2$ will follow $CC_3$. This intuitively leads to $CC_2$ having more followers in expectation than $CC_3$ and fewer than $CC_1$. For simplicity, we will only formalize this intuition for $n=2$:

$$\begin{aligned} {\mathbb {E}}[a^{\infty }_{., 1}]&= \sum _{k=0}^m {\mathbb {P}}(a^1_{., 1} = k) \cdot {\mathbb {E}}[a^{\infty }_{., 1}| a^1_{., 1} = k] = \sum _{k=0}^{\left[ \frac{m-1}{2} \right] } \frac{\left( {\begin{array}{c}k\\ m\end{array}}\right) }{n^m} \cdot k + \sum _{k = \left[ \frac{m-1}{2} \right] +1}^{m} \frac{\left( {\begin{array}{c}k\\ m\end{array}}\right) }{n^m} \cdot m. \end{aligned}$$

As the sum is larger than $${\mathbb {E}}[a^{\infty }_{., 2}]= \sum _{k = 0}^{m} \frac{\left( {\begin{array}{c}k\\ m\end{array}}\right) }{n^m} \cdot k$$, $(A_\infty ^t)_{t\ge 0}$ is ex-ante fair.

Similarly, for extreme anti-PA, the observation implies that whenever a $CC_i$ is the *CC* with the least number of followers, they will be followed by at least as many *CC*s as when $CC_j$ of lesser quality is recommended. For $n=2$, (a) if $X_1 \supset X_2$ then none of the two CCs will change their follower count, and (b) if $X_2 \supseteq X_1$ then eventually all seekers will follow $CC_1$ and $CC_2$ will remain with the followers in $X_2$. Thus, ${\mathbb {E}}[a^{-\infty }_{., 1}] \ge {\mathbb {E}}[a^{-\infty }_{., 2}]$ and the process is ex-ante fair when there are two CCs. The same intuition can be used to show the result for general values of *n*. $\square$

## Simulation results

Our theoretical results showed that while individual fairness with respect to the expected number of followers is guaranteed, many realized outcomes could be unfair. In this section, we use simulations for a more granular understanding of the role of the recommendation process in the network formation. More precisely, we look at how it impacts (a) the structure of the follower network, (b) the chances of fair outcomes for content creators (CCs), and (c) the satisfaction of seekers. Our theoretical analysis also reveals that while extreme recommendation processes are expected to converge quickly, others could take long periods. As such, our simulations will look beyond fairness at convergence, thus also investigating the effect of time.

*Virtual Experiment Design.* We run our simulations for a platform with 100 CCs and 10000 seekers for different recommendation processes (values of $\alpha$) and 1000 iterations^12^. Since recommendation processes are based on random functions, we also simulate each parameter configuration with 1000 different random seeds. We chose this number to balance the quality of results with the run-time and memory tractability. During the analysis, we investigate ex-post individual fairness and user satisfaction at different timesteps. We use the metrics introduced in the Model Section. For the figures in the main text, we do not include confidence intervals (for clarity). However, alternative visualization with the $95\%$ confidence intervals (CIs) obtained via bootstrapping are included in the Appendix. We will say that differences between two values are significant if their CIs do not overlap. The code is publicly available on our GitHub repository^13^.

### The evolution of the follower network depends on the recommendation process

**Fig. 6:**
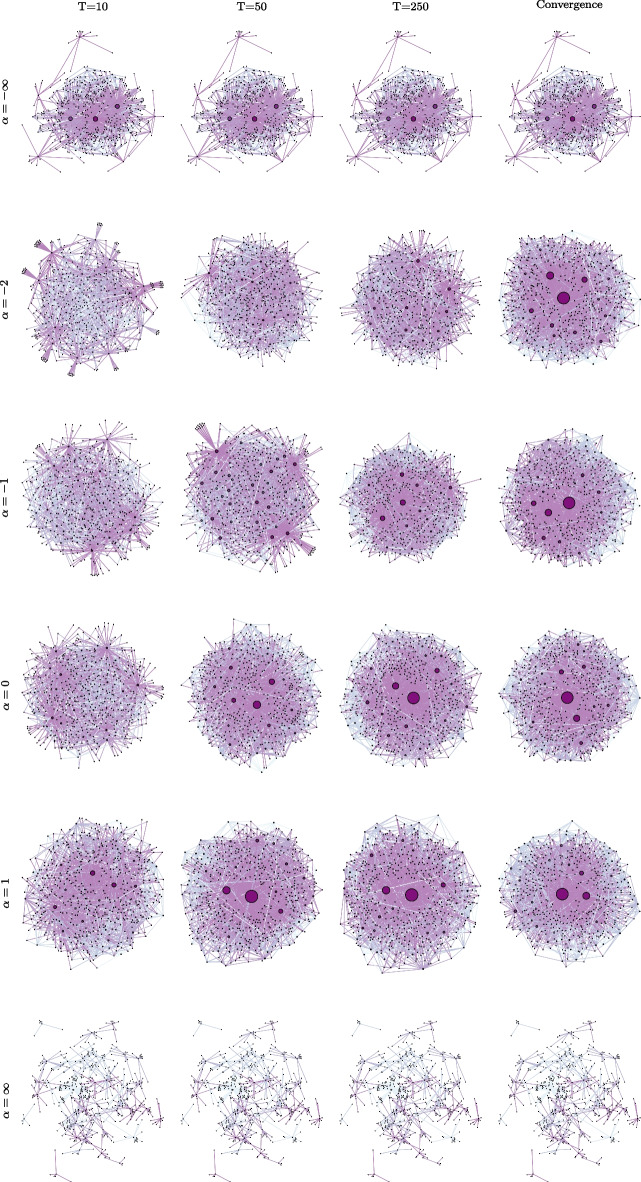
For different levels of $\alpha \in \{-\infty , -2, -1, 0, 1, \infty \}$, we simulate one instance of the network formation model ($m=500$ and $n=50$) and we plot the network at different stages: $T=10, 50, 250$, and at convergence. The size of the nodes is proportional to their in-degree, and the color intensity to their quality

We start our simulation analysis by providing an example of how the network formation process evolves under the different recommendation processes. To do so, we simulate our model under different values of $\alpha \in \{-\infty , -2, -1, 0, 1, \infty \}$. For each scenario, we plot the network after 10, 50, 250 iterations, and at convergence. As shown in Fig. 6, extreme recommendation functions ($\alpha \in \left\{ \pm \infty \right\}$) converge faster, namely in less than 10 iterations, but also lead to sparser networks. As a consequence of the AntiPA-like process, negative but finite values of $\alpha$ are the slowest to reach convergence and lead to very dense networks. More generally, in all scenarios but the Extreme PA, we observe a correlation between the number of followers (which is proportional to the nodes’ size) and their quality (which is proportional to the nodes’ color intensity). A closer look shows that neither Extreme PA nor Extreme AntiPA leads to $CC_1$ fairness. Yet, Extreme AntiPA achieves a higher level of fairness compared to Extreme PA, with the most followed nodes being of high quality (yet, not being the highest-ranking nodes).

### Slight increases in the visibility of low-popularity CCs improves fairness

**Fig. 7:**
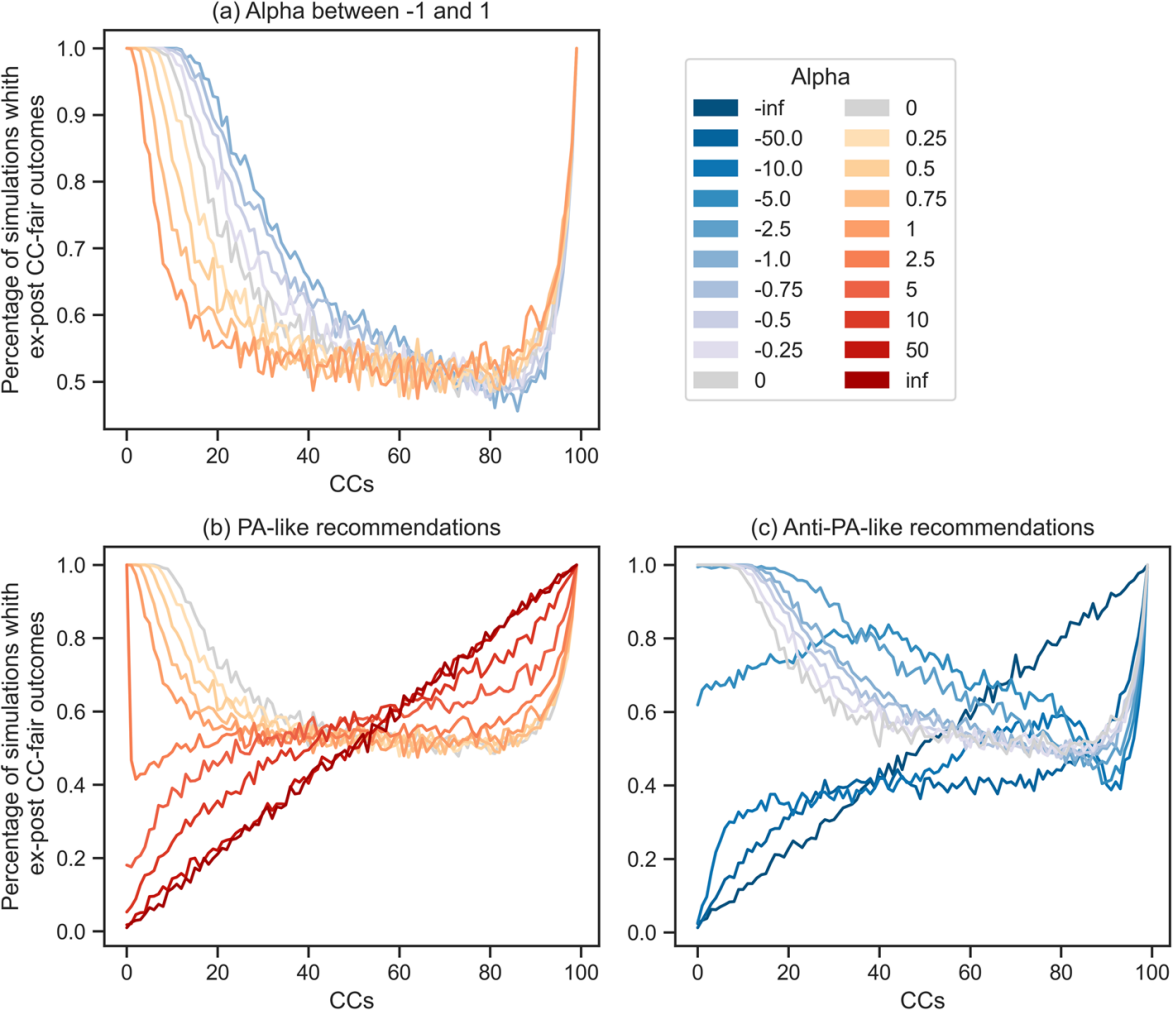
The plots show how different recommendation processes influence the chance of observing fair outcomes for different CCs. The *x*-axis is for CCs (from highest-quality, $CC_1$, to lowest-quality, $CC_{100}$) while the *y*-axis is for the percentage of simulations which were $CC_i$-fair is satisfied after 1000 timesteps. **a** focuses on the effect of increasing the visibility of low-popularity CCs when starting from the realistic recommendation process (i.e., PA with $\alpha = 1$). **b** (respectively, (**c**)) look at the effect of further increasing (respectively, decreasing) the visibility of the most popular CCs

We then closely investigate the effect of reducing popularity bias and even introducing low-level anti-popularity bias on the chance of individually fair outcomes for content creators (CCs). Figure 7a shows the percentage of ex-post fair results for different CCs starting from a realistic PA (with $\alpha = 1$ (Pagan et al. 2021)) to AntiPA (with $\alpha = -1$). In accordance with prior work, our results confirm that popularity biases negatively affect CC-discoverability. Fairness-wise, this leads to significant chances of unfair outcomes for most CCs (see Fig. 11a in the Appendix). More precisely, as the recommendation process relies more and more on popularity, increasingly many CCs need an important degree of luck to obtain a fair outcome, since for many CCs, the chance of arriving at a fair outcome is around $50\%$.

Our simulations also show that interventions that slightly increase exploration by giving higher visibility to low-popularity CCs increase fairness for the top-quality CCs. This increase can be observed for most of the 75% top-quality CCs, and is significant for the top 50%. However, this gain is obtained by a slight decrease in the chances of fairness for bottom-quality CCs.

### High levels of visibility of either low or high-popularity CCs reduces fairness

Our theoretical results show that extreme recommendations have low chances of fairness for top-quality CCs. As such, increases in popularity or anti-popularity biases (i.e., of $|\alpha |$) leads PA and AntiPA processes to approach their extreme versions and reduce fairness at the top. We verify this intuition and investigate the rate of change.

As shown in Fig. 7b and c, large (anti-)popularity biases do indeed exacerbate the chance of unfairness for top-quality CCs. With the increase in $|\alpha |$, PA-like processes increase fairness guarantees for the bottom-quality 50% CCs at the expense of the top-quality CCs. Contrary, AntiPA continues to increase fairness, especially for middle-quality CCs. However, very large $|\alpha |$ ultimately leads to lower fairness chances for all CCs. This suggests that exploration should be carefully introduced, as too much could easily harm up to all CCs.

### Fairness improves throughout time

The fact that outcomes can be $CC_1$-unfair (see Fig. 7b and  c) could seem contradictory to the theoretical results. While we proved that the system was always ex-post $CC_1$-fair at convergence when availability was guaranteed, simulations show that this is not necessarily the case after 1000 timesteps. Therefore, the most likely explanation is the lack of convergence within the given timeframe. We further investigate the convergence of the given processes and the effects of time constraints on the observed outcome.

**Fig. 8:**
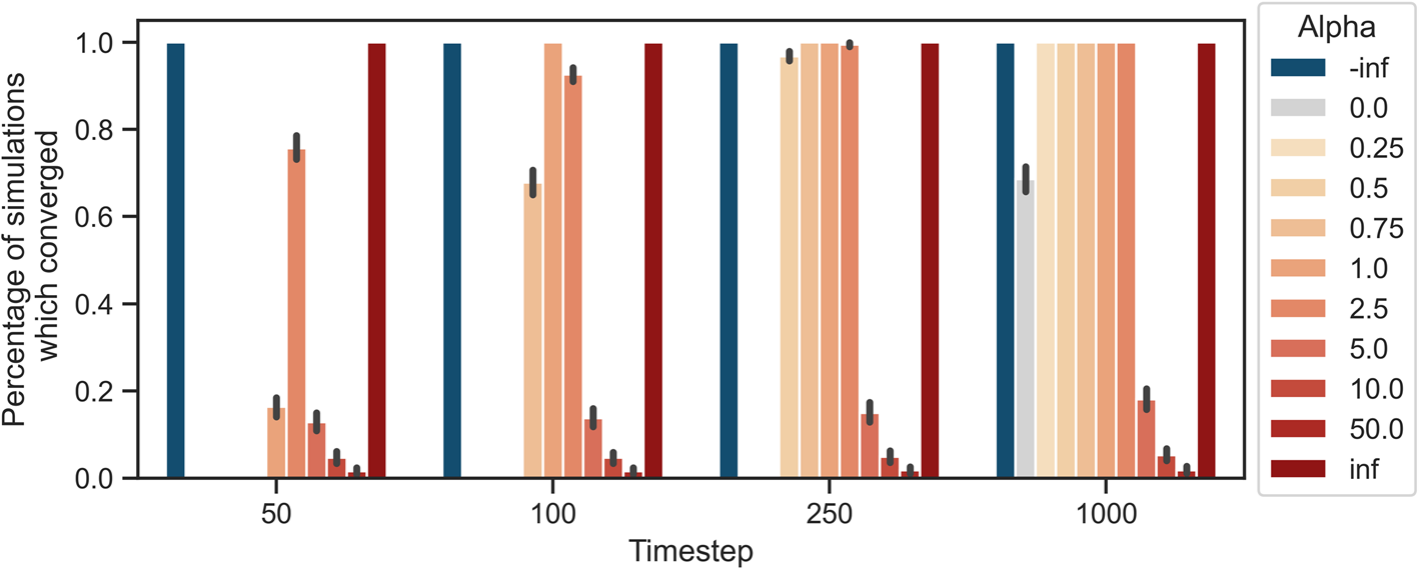
The plot shows the percentage of simulations that converged within a given number of timesteps. We show the results for different values of $\alpha$. We exclude finite and negative values of $\alpha$ as they never converged with 1000 timesteps

Figure 8 confirms both our intuition and the theoretical analysis. In accordance to Theorems 4 and 7, the extreme recommendation processes always converge. However, this was not the case for the rest. In the near and short future (i.e., within 50 or 100 timesteps) most of the recommendation processes rarely converge. While longer timeframes obviously increase the percentage of simulations that converge, PA-like processes with large values of $\alpha$ still have low chances of convergence. Moreover, none of the non-extreme anti-PA processes ($0> \alpha \ne - \infty$) converge within 1000 timesteps. We thus omit them from Fig. 8. Altogether these results underline the importance of analyzing fairness in transient states, especially for the many processes with long expected times to absorption. Additionally, together with Fig. 7b and c it signals that short and long-term fairness could be significantly different.

**Fig. 9:**
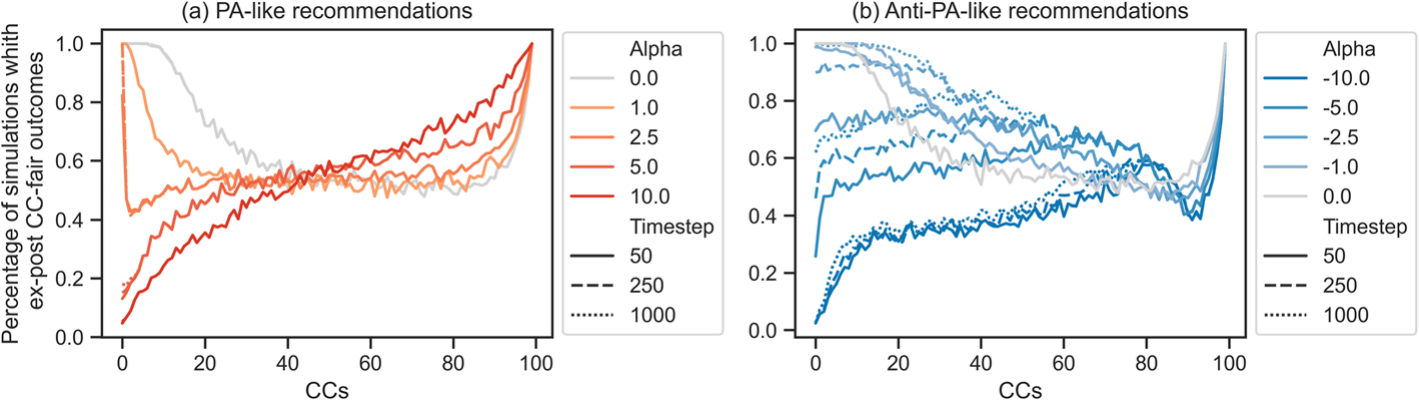
The plot shows the impact of the running time on the chances of observing fair outcomes for different CCs in two scenarios: **a** PA-like and **b** Anti-PA-like recommendation processes

In Fig. 9 we look at the impact of the number of timesteps on individual fairness. For UR and PA-like recommendations, the chance of fair outcomes is independent of the number of timesteps for most CCs (the only exception being the top-quality CC). Conversely, anti-PA has significant differences in the likelihood of fair outcomes depending on the number of timesteps, especially for the 25% top-quality CCs (see Fig. 12 in the Appendix for more information). This implies that different recommendation processes could be more appropriate depending on the time horizon in which we want to minimize the impact of luck (i.e., maximize fairness) and for whom. While implementing anti-PA does improve long-term fairness, only the middle-quality CCs will benefit in the short term. For the top-quality ones, it does significantly worse.

### Seekers are most satisfied under PA with medium importance to popularity

**Fig. 10:**
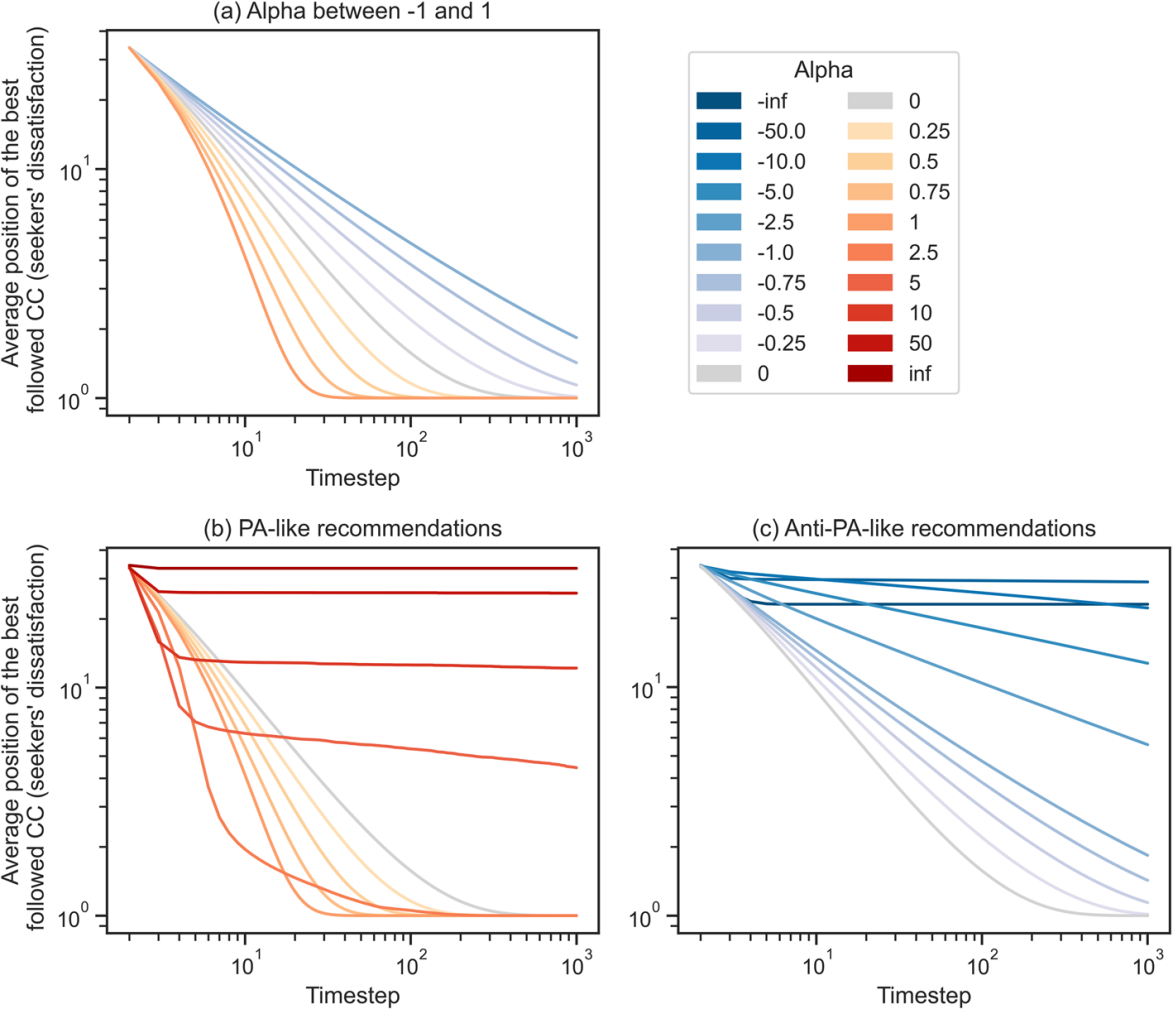
Log-log plots showing how different recommendation processes influence the satisfaction of seekers over time. We use the *x*-axis for the timestep and the *y*-axis for the average quality-position of the most followed CC (averaged over both users and simulations). Low *y*-values, thus, correspond to higher levels of user satisfaction. As before, subplot **a** focuses on the effect of increasing the visibility of low-popularity CCs when starting from the realistic recommendation process (i.e., PA with $\alpha = 1$), while subplots **b** and **c** look at the effects of further increasing or respectively decreasing the visibility of the most popular CCs

**Fig. 11:**
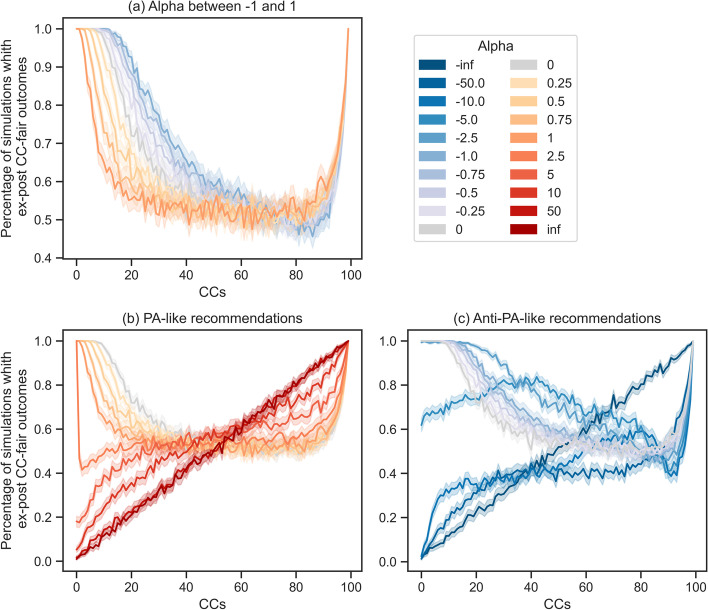
This is an alternative version of Fig. 7 in which $95\%$ confidence intervals are displayed. The plots show how different recommendation processes influence the chance of observing fair outcomes for different CCs. The *x*-axis is for CCs (from highest-quality, $CC_1$, to lowest-quality, $CC_{100}$) while the *y*-axis is for the percentage of simulations which were $CC_i$-fair after 1000 timesteps. **a** focuses on the effect of increasing the visibility of low-popularity CCs when starting from the realistic recommendation process (i.e., PA with $\alpha = 1$). **b** and **c** look at the effects of further increasing or respectively decreasing the visibility of the most popular CCs

**Fig. 12:**
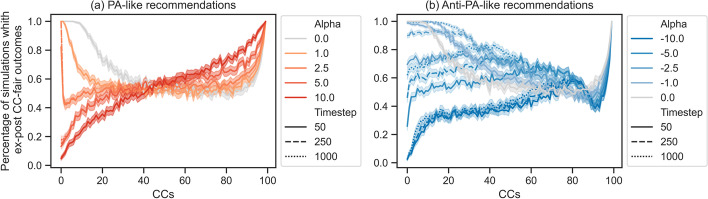
This is an alternative version of Figure 9 in which $95\%$ confidence intervals are displayed. This figure shows how the chances of observing fair outcomes for different CCs change thought time for different **a** PA-like and **b** Anti-PA-like recommendation processes

Finally, we investigate the effect of popularity biases on the satisfaction of seekers. Figure 10 shows how user dissatisfaction changes over time depending on the recommendation process. As expected, seekers become more satisfied as time passes. By comparing with prior results, we notice that seekers and CCs benefit from different recommendation processes: While most CCs would prefer reduced to low anti-popularity biases (Fig. 7a), seekers are less satisfied with such changes (Fig. 10a). In fact, seekers are, on average, the most satisfied with PA-like processes with $\alpha$ around 1. However, if we extend the comparisons towards extreme processes, we can see that large absolute values of $\alpha$ also harm seeker satisfaction.

This shows that while platforms do not benefit from introducing large levels of popularity biases, low levels could improve the satisfaction of their consumers. Moreover, when seekers are solely interested in finding the best creators, platforms could be harmed if they introduce anti-PA recommendation processes.

## Conclusion

This paper investigated the effects of recommendation popularity biases on the individual fairness of content creators (CCs). To do so, it (a) extended prior network models with a parametrized recommendation function with popularity and anti-popularity biases, (b) defined two types of individual fairness measures (ex-ante and ex-post), and (c) defined a measure of user satisfaction. We explored the properties of this model both analytically and through simulations. The theoretical analysis revealed that the network evolution over time is an absorbing Markov Chain, where the probability of transitioning between states varies much depending on the level and type of popularity bias. Importantly, we proved that the accessibility of CCs to seekers is critical in guaranteeing fair outcomes for CCs: While under accessibility, all the absorbing states are ex-post fair for the best-quality CC, this is rarely the case for extreme recommendation processes. Such extreme processes do, however, continue to be ex-ante fair for the top-quality CC, thus proving we should look beyond fairness in expectation when analyzing CC-centered platforms. Moreover, we showed that extreme processes are expected to converge quickly, thus putting them in stark contrast with non-extreme alternatives.

The simulation results brought a more complete and granular understanding of how popularity biases affect the users and whether anti-popularity could help overcome those biases. First, they revealed that decreasing popularity biases and even introducing low anti-popularity biases helps reduce the impact of luck in the fairness of the outcome for most CCs. However, too much visibility of low-popularity items can negatively impact the chances of ex-post fairness, especially for the top-quality CCs. This is mainly caused by realistic time constraints, as more exploration of unpopular CCs requires increased search times for seekers. In fact, quality-oriented seekers are the most satisfied under recommendation processes with medium popularity biases. From there, larger importance of popularity or introducing anti-popularity biases boost their dissatisfaction.

Altogether our results indicate that the optimum with respect to both seeker satisfaction and time to convergence is for a preferential attachment (PA)-like process. However, CCs have more chances of being treated fairly under anti-PA recommendations. Thus, in essence, decreasing the level of popularity biases in recommendations trades the satisfaction and search time of seekers for more probable CC-fair outcomes. This makes the intervention of introducing anti-popularity biases unlikely to be introduced by platforms. Moreover, extreme care is needed even if platforms decide to implement such an intervention: The optimum level of bias depends largely on when they want to improve fairness and for whom.

Before concluding this final section of the paper, we want to underline what we believe to be the main limitations of our work, paired with the most promising directions for future work. At the core of the enclosed research lies the simplicity of the model, which presents itself both as an opportunity and a limitation. While some level of simplicity is both inevitable for a feasible theoretical analysis and valuable in preserving the interpretability of simulation results, real-world systems do sometimes depart from our assumptions. Three such examples are assuming that (a) CCs can be described through a single dimension (i.e., an ordinal attribute that we call quality), (b) seekers exhibit a consistent and unanimous quality-based decision function, and (c) the recommender system is solely based on popularity. As argued by prior work (Pagan et al. 2021), the resulting model is simple (as it models one community of users with an agreement and consistency in content evaluation) yet confirmed by data to produce realistic outcomes. We persisted with these assumptions in order to isolate and better understand the individual role of popularity biases in recommendations and moderation in the observed lack of individual fairness. More precisely, thanks to this simplicity, we showed that even when all users agree on their evaluation of CCs, for many CCs it is still a matter of luck whether or not they reach a fair outcome. The lack of fairness guarantees is thus not the sole byproduct of a complex system characterized by audiences with various tastes whose attention is steered by complicated recommender systems. Instead, it is stemming from the randomness in the initial phases of the exploration process and is possibly exacerbated by the exploitation in PA-like network formation processes.

Future work could thus extend the model to account for multi-dimensional attribute spaces, which contain not only ordinal but also nominal attributes (Spiller and Belogolova 2017) (e.g., being serious versus funny, the gender or race of CCs). Moreover, extensions might target the decision-making function of seekers, which could differentiate in taste by making noisy decisions, weighting attributes differently, or showing various levels of trust in recommendations. Such an analysis would help us better understand the interplay between different communities of possibly disproportionate sizes (e.g., creators and seekers of different music genres). The plurality of attributes also opens the door to investigating personalized recommendation processes (e.g., collaborative filtering), different notions of fairness (e.g., group fairness), and non-static seeker preferences (e.g., changes in taste through user inertia). When it comes to interventions, additional work could aim beyond the goal of understanding the effect of popularity biases with unique recommendations and look at more nuanced approaches proposed for amortizing fairness over time (Biega et al. 2018). In short, recommender systems and moderation are manifold techniques embedded within complex sociotechnical systems, and our work is just one piece of this elaborate puzzle. Although many questions remain unanswered, we believe our analysis bears one cornerstone message: Even when all seekers agree on their evaluation of CCs there are still significant chances that outcomes will not be fair for many CCs, and if we want to lower these chances, we must encourage the exploration of unpopular CCs. Yet, the benefit will become visible only after a sufficiently large time horizon.

## Figures and Tables

**Fig. 13:**
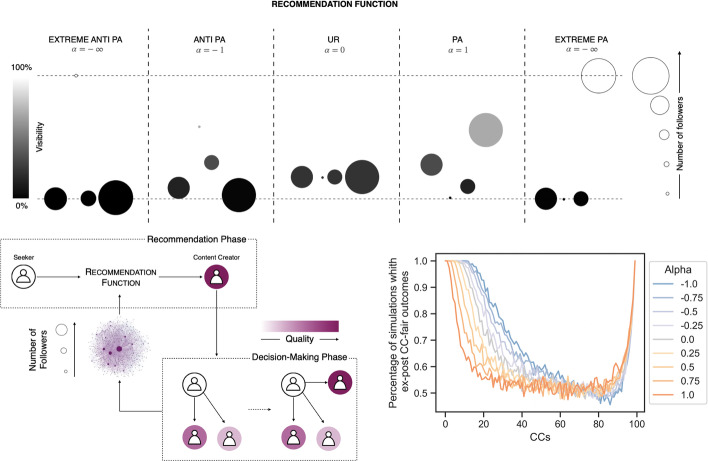
Graphical abstract. This figure shows a summary of our paper, as it includes **a** a representation of the type of recommendation functions resulted from each recommendation process (top), **b** an overview of the agent-based model (bottom left), and **c** a plot for the probability for each *CC* of achieving a fair outcome under different recommendation processes (bottom right)

## Data Availability

The datasets used and/or analyzed during the current study are available from the corresponding author upon reasonable request. The code to generate the data and analyze it can be found on our GitHub repository (https://github.com/StefaniaI/ABM-IFforSMI). The generated synthetic data has more than 145GB. Due to its size, we do not provide the synthetic data directly. However, the instructions to re-generate it can be found in the aforementioned repository. Project name: ABM-IFforSMI. Project home page: https://github.com/StefaniaI/ABM-IFforSMI. Operating system(s): Platform independent. Programming language: Python. Other requirements: Python 3.8.1 or higher. License: GNU General Public License 3.0.

## Appendix

### Figure alternatives with $95\%$ confidence intervals

Figure 11 is an alternative of Fig.  7 which also contains the CIs obtained via bootstraping (1000 bootstraps and $95\%$ CI). Similarly, Fig. 12 is an alternative of Fig. 9, which also contains the CIs similarly obtained. We do not make such an alternative for Fig. 10 as CIs are so small that they are mostly indistinguishable. Finally, Fig. 13 shows our graphical abstract.

## Abbreviations

CC: One content creator (i.e., a user on the platform who generates content)
RS: Recommender system
PA: Preferential attachment
MC: Markov Chain
$anti-PA$: Anti-preferential attachment
CI: Confidence interval
iff: If and only if
s.t.: Such that

## Footnotes

1 This paper is an extension of our prior work (Ionescu et al. 2023). It is thus important to note that the model, fairness metrics, and theoretical results are similar to the ones in the aforementioned paper. However, the current work brings significant novelty. Regarding the model and metrics, we parameterized the recommendation function (thus allowing us to investigate recommendations with $\alpha$ values different from 0, 1, and $\infty$ – i.e., UR, PA, and ExtremePA in Ionescu et al. (2023)) and added a metric for user satisfaction. In the theoretical analysis, we generalized the results to arbitrarily finite values of $\alpha$. We also added theoretical results for $\alpha = - \infty$ (Extreme anti-PA). Finally, we developed the code (publicly available at https://github.com/StefaniaI/ABM-IFforSMI) and run simulations for different scenarios. This gives a complete picture of the role of popularity biases on the different stakeholders.

2 Theories and other models often distinguish between two types of attributes that ultimately count within the decision-making process of users: (a) ordinal or competing (i.e., attributes on which others prefer a value as large as possible, such as sound quality), and (b) nominal or matching (i.e., attributes on which others prefer a value matching their own, such as being funny or serious) (Spiller and Belogolova 2017; Liu et al. 2020; Ionescu et al. 2021; Bruch and Newman 2018).

3 While in general seekers could create content and CCs can also follow other CCs, we make this simplifying assumption as: (a) in practice, only a small share of users create content (Nielsen 2006), so (b) relatively few edges would be between CCs. A more detailed discussion on modelling assumptions can be found in prior work (Pagan et al. 2021; Ionescu et al. 2023).

4 Although, in general, each seeker receives a list of recommendations, for simplicity and to allow comparable results with prior work (Pagan et al. 2021), we provide only one recommendation for each seeker per timestep.

5 As usual, we use infinity as a shorthand notation for the limit. For example, ${\mathbb {P}}(R^t_{\infty } (s) = i) = \lim _{\alpha \rightarrow \infty } \frac{(1+a^t_{., i})^{\alpha }}{ \sum _{j\in \overline{n}} (1+a^t_{., j})^{\alpha }}$.

6 This definition is slightly simplified from prior work. (Dean et al. 2020) say that an item is reachable by a user if the user could change their interaction history s.t. the item in question could be recommended to them. Since seekers are many and have identical preferences, unilateral changes in follower decisions have limited to no impact on recommendations. As such, for our purpose, the two definitions are almost always equivalent.

7 This definition does not consider ties as a signal for unfairness and, thus, represents a weak definition of individual fairness. As we will show in the theoretical results, this is not a key assumption, as ties in follower counts are rare when the number of seekers is large.

8 These first two results do in fact hold for any value of $\alpha$.

9 Perhaps the easiest is to show this or $c= 4n/(n-1)$. Using Lemma 2 we can prove that $p_{B^*, S\cup S^*}\ge 1/2n$ (although better bounds can be obtained).

10 By definition, a function *f*(*x*) is asymptotically equivalent to a function *g*(*x*) if $lim_{x\rightarrow \infty } \frac{f(x)}{g(x)} =1$. Note that since, by Lemma 3, the chance of equalities in the number of followers after the first timestep goes to zero as the number of seekers goes to infinity, Theorem 6 implies the process is almost always expected to converge within about $\frac{n-1}{n}\cdot e$ under extreme anti-PA.

11 “Although, on average, quality is positively related to success, songs of any given quality can experience a wide range of outcomes”s (from (Salganik et al. 2006), page 855).

12 In the network analysis in (a), we use $n=50$ CCs and $m=500$ seekers to enhance visualization clarity.

13 GitHub link for the code: https://github.com/StefaniaI/ABM-IFforSMI.
